# Measuring the Head Circumference on MRI in Children: an Interrater Study

**DOI:** 10.1007/s00062-021-01019-z

**Published:** 2021-05-21

**Authors:** Alexander Rau, Theo Demerath, Nico Kremers, Matthias Eckenweiler, Rieka von der Warth, Horst Urbach

**Affiliations:** 1 Dept. of Neuroradiology, University Medical Center Freiburg, Freiburg, Germany; 2 Dept. of Neuropediatrics and Muscle Disorders, University Medical Center Freiburg, Freiburg, Germany; 3 Institute of Medical Biometry and Statistics, Section of Health Care Research and Rehabilitation Research, Medical Center – University of Freiburg, Faculty of Medicine, University of Freiburg, Freiburg, Germany

**Keywords:** Microcephaly, Megalocephaly, Cephalometry, Macrocephaly, Neuropediatrics

## Abstract

**Purpose:**

The head circumference is typically used as a surrogate parameter for the development of the central nervous system and intracranial structures and is an important clinical parameter in neuropediatrics. As magnetic resonance images (MRI) can be freely zoomed, visual analysis of the head size often relies on impressions, such as the craniofacial ratio or a simplified gyral pattern. Aim of this study was to validate an MRI-based method to measure the head circumference.

**Methods:**

Head circumferences of 85 children (41 microcephalies, 22 macrocephalies and 22 normal controls; 47 male, mean age 3.22 ± 2.45 years, range 0.19–10.42 years) were retrospectively measured using sagittal 3D-T1w (MPRAGE) data sets. Three readers independently placed an ovoid region of interest in an axial plane starting from the supraorbital bulge and covering the largest supra-auricular head circumference. Clinical measurements of the head circumference taken within an acceptable period served for comparative purposes. Reliability was assessed by calculating the total error of measurement (TEM) and the intraclass correlation coefficient (ICC).

**Results:**

A close correlation was found between MRI-based and clinical measurements. The interrater reliability was excellent (ICC 0.985, 95% confidence interval 0.952–0.993). Absolute TEM ranged from 0.47–0.75, resulting in relative TEM ranging from 1.0–1.6%. Thus, TEMs were classified as acceptable. The mean accuracy of MRI-based measurements was high at 0.94.

**Conclusion:**

The head circumference can be reliably determined with a simple measurement on 3D sequences using multiplanar reformations. This approach may help to diagnose microcephaly and macrocephaly, especially when the head circumference is not reported by the referring physician.

## Key Points

- Head circumference was measured based on 3D T1-weighted magnetic resonance imaging (MRI) datasets.
- Retrospective interrater study showed close correlation of clinical and radiological measurements.
- Radiologically measured head circumference showed high accuracy and reliability.

## Introduction

The head circumference (HC) is a validated parameter of a pediatric examination. It is easily measured and used as surrogate parameter for the development of the central nervous system and intracranial structures [1].

The circumference of the skull starting from the supraorbital bulge is determined using a measuring tape [2, 3]. Age-specific percentile curves are derived from large data collections [3]. Deviations of more than two standard deviations are considered as abnormal: HC values below the 3rd percentile define microcephaly and above the 97th macrocephaly [4, 5].

Microcephaly affects approximately 1.6/1000 live births and is often associated with a developmental delay [6]. Other common comorbidities include epilepsy, cerebral palsy, and mental retardation. The causes include genetic syndromes, environmental toxins, infectious diseases and structural brain disorders [7].

Macrocephaly affects up to 5% of pediatric patients and is often caused by a disturbance in the cerebrospinal fluid (CSF) circulation [8] while genetic syndromes are associated with macrocephaly, too [9].

Currently, there are only limited data on the correlation between HC and imaging features of microcephaly/macrocephaly and associated cerebral malformations [10–14].

If a pathological HC is documented, magnetic resonance imaging (MRI) is recommended to further evaluate the intracranial structures and investigate possible underlying pathologies [15].

**Fig. 1:**
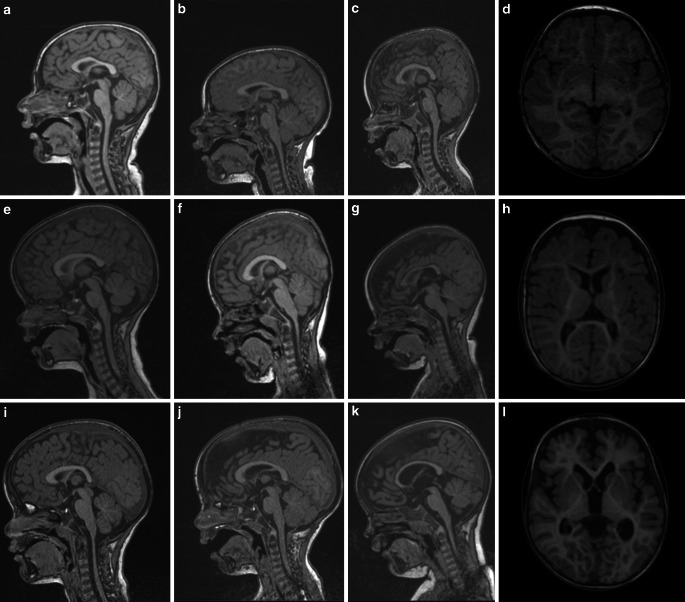
Midsagittal reformats of 3D T1-weighted MPRAGE (Magnetization Prepared – RApid Gradient Echo) sequences with FOV height of 25 cm. As children have different ages (10 months to 3 years) and FOVs are adapted to the head sizes as depicted, it is difficult to assess which children are microcephalic, have a normal head size or are macrocephalic. Microcephaly: **a** male, 3 years, 47 cm HC (0.7 cm < 3rd percentile), **b** male, 4 years, 47 cm HC (1.2 cm below the 3rd percentile), **c** male, 15 months, 43 cm HC (1 cm < 3rd percentile). Normal HC: **e** female, 3 $\frac{10}{12}$ years, 51.5 cm HC (92nd percentile), **f** male, 3 $\frac{4}{12}$ years, 50 cm HC (24th percentile), **g** male, 10 months, 45 cm HC (33rd percentile). Macrocephaly: **i** male, 3 $\frac{10}{12}$ years, 53 cm HC (0.5 cm > 97th percentile), **j** male, 3 $\frac{4}{12}$ years, 56.2 cm HC (2.8 cm > 99th percentile), **k** female, 24 months, 51 cm HC (0.5 cm > 97th percentile). By measuring the head circumference, which is exemplary shown in the right-hand column, a child’s head size can be easily classified: **d** (case **c**) clinical HC 43 cm, MRI-based HC 435 mm, **h** (case **g**) clinical HC 45 cm, MRI-based HC 440 mm, **l** (case **k**) clinical HC 51 cm, MRI-based HC 505 mm

Since the HC is not always documented by referring physicians, radiologists might feel the need to measure the HC using MRI; however, it is difficult to assess the head size on MRI as the head size is age-dependent and the field of view (FOV) on MRI is typically adapted to the head size in order to increase the spatial resolution, but different FOV are not apparent. Positioning of the head within the head coil may be variable, and different reformations of the increasingly acquired 3D sequences make the reader’s interpretation difficult (see Fig. 1 for illustration). Thus, assessment of the head size is often limited to the craniofacial ratio while objective measurements are lacking. The craniofacial ratio is defined as the ratio of the area of the intracranial structures to the area of the face on midsagittal reconstructions. While only subjective values can be evaluated and no normal values exist, it is known to be large at birth and decreases with increasing age [10].

Knowledge of the HC is essential and might facilitate evaluation of pediatric brain imaging; however, repeated clinical tape measurement is not feasible during clinical routine, emphasizing the need for a simple MRI-based method to measure the HC.

Thus, the purpose of this retrospective study was to develop a simple MRI-based measurement of the head circumference and to validate it by comparing MRI-based with clinical measurements in pediatric patients.

## Methods

This trial was carried out according to the guidelines for reporting reliability and agreement studies [16]. This study was performed in line with the principles of the Declaration of Helsinki. Approval was granted by the local ethics committee, informed written consent was waived.

## Assessment of HC

We retrospectively included patients who had been admitted to the department of pediatrics and underwent cranial MRI with a sagittal 3D MPRAGE (Magnetization Prepared – RApid Gradient Echo) sequence using a 1.5 T Siemens Avanto with a 12-channel head coil and a 3 T Siemens Trio scanner (Siemens Healthcare, Erlangen, Germany) with a 32-channel head coil, respectively. Patients had to be younger than 18 years and a clinically documented HC within an acceptable period before or after the MRI study measured by an experienced clinician was necessary. In order to take the age-dependent dynamics of head growth into account, the time intervals between imaging and clinical measurement were limited. Accordingly, for younger patients with relatively faster growth, a shorter time period was chosen. This acceptable period was defined as 3 months for children older than 6 years, as 1 month for children aged 2–6 years and as 1 week for children younger than 2 years. To ensure that a sufficient number of pathological HC were included a selected sampling approach was chosen, initially identifying 20 children with microcephaly and 20 with macrocephaly by a report query. A total of 45 more patients were then included by means of a backward case tracking based on MRI examinations and existing clinical data.

**Fig. 2:**
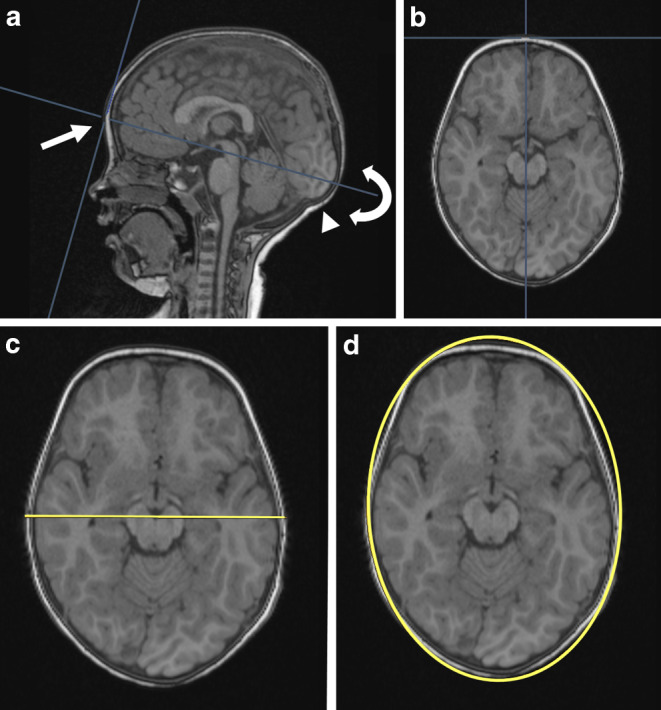
A 2‑year-old girl with microcephaly (clinical head circumference 45 cm/ < 1st percentile) **a** Midsagittal MPRAGE (Magnetization Prepared – RApid Gradient Echo) 3D-reformat with a nearly normal craniofacial ratio. For MRI-based head circumference estimation identify the supraorbital bulge (*white arrow*). Then adapt the axial plane in the 3D-reformation (depicted by the *white curved arrow*) until the largest supra-auricular head circumference is achieved (as depicted in **b**). The inion (*white arrowhead*) can be used as a landmark. **c**, **d** An ovoid ROI is created in axial reformat by first identifying the lateral expansion (*yellow line* in **c**) and then the anteroposterior expansion (**d**). MRI-based head circumference is 45.1 cm

The three readers (6, 4, and 3 years of experience in clinical neuroradiology) independently reviewed the MR images using the in-house Picture Archiving and Communication System (PACS) (IMPAX EE R20 VXII SU4, Agfa HealthCare N.V., Mortsel, Belgium) with application of the 3D reformations plug-in, being blinded for patient age, gender and clinical data. An ovoid region of interest (ROI) was placed in an axial plane starting from the supraorbital bulge and covering the largest supra-auricular HC (Fig. 2). If the head shape was asymmetric, raters were advised to place the ROI as accurate as possible with the aim of reproducing the outer circumference as accurately as possible. Individual values were transformed into percentiles [3]. Following the World Health Organization (WHO) 2016 guidelines, the 3rd percentile was chosen as a threshold for a pathologically small head circumference [4, 5] and the 97th percentile was used for macrocephaly.

All readings were independently performed in summer 2020 at the department of neuroradiology. For training purposes, readers were provided with the clinical HC measurement once the MRI-based measurement was performed in the first nine cases.

## Statistical Analysis

Interrater reliability was assessed using the intraclass correlation coefficient (ICC) based on a two-way random effects model [17]. Also, absolute and relative technical errors of measurements (TEM) (variation of measurements performed by different anthropometrists in the same group of persons) were calculated as proposed by Perini et al. [18]. Accuracy was calculated according to Baratloo et al. [19].

A comparison between the MRI-based and clinical measurements using a measuring tape was done on a descriptive level, testing for linearity using a scatterplot. All analyses were performed using SPSS Statistics 26 (IBM Corporation, Armonk, NY, USA) and Microsoft Excel 2010 (Microsoft Corporation, Redmond, WA, USA).

## Results

### Sample

This study included 85 children, 47 (55.3%) male and 38 (44.7%) female. Mean age at the time of clinical measurement was 3.18 years (standard deviation [SD] 2.45 years, range 0.21–10.77 years) and 3.22 years (SD 2.45 years, range 0.19–10.42 years) at the time of MRI based measurement, respectively. Based on clinical tape measurements, 41 patients were microcephalic (48.2%; of whom 37 were diagnosed with severe microcephaly ≤ 1st percentile), 22 were macrocephalic (25.9%; 16 with severe macrocephaly ≥ 99th percentile) and 22 (25.9%) had a normal HC. The HC ranged from 36–56 cm in both clinical tape measurement and MRI measurement. See Table 1 for details.

**Table 1:** Head circumference values (in cm) determined by clinical tape measurement and MRI-based measurements

	Mean	SD	Range
*Tape measurement*	47.92	4.92	36.5–56.6
*Reader 1*	47.92	4.86	36.4–56.6
*Reader 2*	48.35	4.78	36.7–56.9
*Reader 3*	47.55	4.78	36.7–56.5

*SD* Standard deviation

### Interrater Reliability

Interrater agreement was excellent with an ICC of 0.985 (95% confidence interval 0.952–0.993). Absolute TEM ranged from 0.47–0.75, resulting in relative TEM ranging from 1.0–1.6%. Thus, TEMs were classified as acceptable.

The TEM are shown in Table 2, classification was based on that of Perini et al. [18].

**Table 2:** Technical error of MRI-based measurements between readers

Reader	Absolute TEM	Relative TEM (%)	Classification
*1–2*	0.52	1.1	Acceptable
*1–3*	0.47	1.0	Acceptable
*2–3*	0.75	1.6	Acceptable^a^

*Classification based on Perini et al. *[18]

*TEM* technical errors of measurements

^a^Acceptable for readers at beginner level

### Accuracy of MRI-based Measurement

Table 3 shows the accuracy of the MRI-based measurement, comparing it to the clinical tape measurement.

**Table 3:** Agreement between individual MRI-based ratings and tape-based measurement of head circumference

		Rater 1			Rater 2			Rater 3			MRI-based mean		
		Micro	Norm	Macro	Micro	Norm	Macro	Micro	Norm	Macro	Micro	Norm	Macro
*Tape measurement*	*Micro*	38	3	0	35	6	0	41	0	0	41	0	0
	*Norm*	1	20	1	0	20	2	3	18	1	0	20	2
	*Macro*	0	3	19	0	3	19	0	8	14	0	3	19

The count of the respective allocations are indicated.

*Micro* microcephalic, *Norm* normocephalic, *Macro* macrocephalic

Rater 1 correctly identified 57 of 63 pathological HCs and 20 of 22 normal HCs. Rater 2 correctly assessed 54 pathological and 20 normal HCs and rater 3 identified 55 pathological and 18 normal HCs. Rater 2 missed 5 cases of severe microcephaly and rater 3  cases of severe macrocephaly as normal HC, respectively. In those falsely classified cases, the MRI-based percentiles determined were close to the pathologic values. One case (male, 42 months old, clinical measurement 4 weeks prior to imaging) with a clinical measurement of 54 cm referring to the 99th percentile was missed by all raters (51.5 cm/69th percentile, 52.5 cm/90th percentile and 51.0 cm/54th percentile). Another case was also falsely classified as normal by all raters: male, 10 years old, clinical measurement on the day of MRI with clinically 56.6 cm HC referring to the 98th percentile and MRI-based 55.4 cm, 54.9 cm and 55.4 cm.

Overall, the sensitivity of the MRI-based measurements was 0.97, specificity 0.87, and accuracy 0.94.

**Fig. 3:**
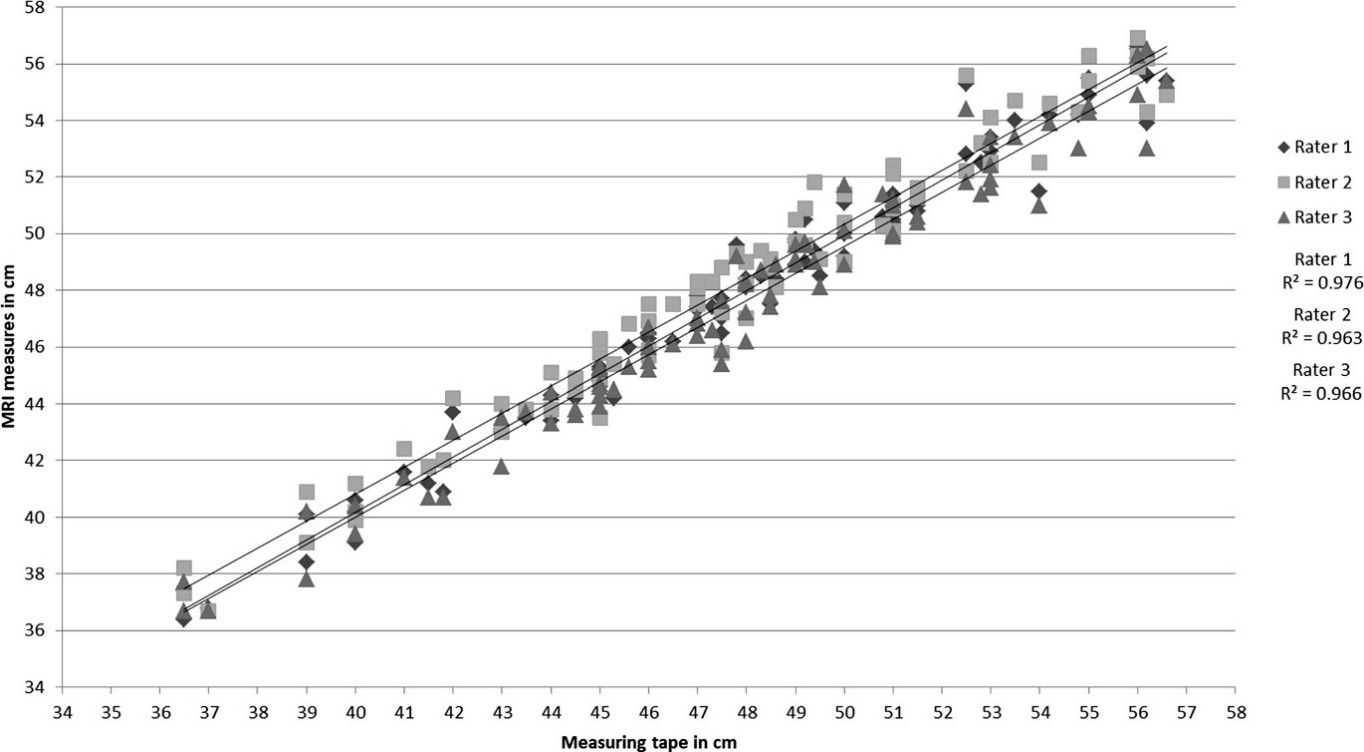
Correlation of absolute clinical tape-based (X axis; in cm) and MRI-based (Y axis; in cm) HC measurements

Testing for linearity using a scatter plot, the regression models showed strong relationships between clinical measurements of MRI measurements. The linear correlation between clinical and MRI-based measurements is shown in Fig. 3.

## Discussion

We evaluated an MRI-based HC measurement method compared it to tape measurement, being validated on both normal and pathological HC. We present a simple, MRI-based algorithm to measure the head circumference (HC) and prove its accuracy by comparing MRI-based and tape measurements typically performed but often not reported by the referring physicians. The measurement requires acquisition of an ideally isotropic 3D sequence. Axial reformats are generated, covering the supraorbital bulge, for identification of the largest head circumference (Figs. 1 and 2).

Approaches on image-based HC estimation have so far been poorly validated. For instance, Smith et al. developed an automated, CT-based approach in which they derived the HC from a bone segmentation [11], while Vorperian et al. evaluated image-based measurements using two orthogonal lines at a level comparable to the clinical measurement to estimate HC [12]. In this latter approach, atypical head shapes were not taken into account, which might be disadvantageous, e.g. in the occurrence of craniosynostosis. Previous studies used clinically assessed HC in children with microcephaly and adults, which were correlated with intracranial volumes without radiologically verifying the clinical measurement [12, 14]. Another study approximated pathological head sizes by examining sagittal 2D images in microcephaly patients [10]. Without clinical information regarding the HC, images were evaluated concerning the craniofacial ratio and the microcephaly was classified into three severity levels using age-matched controls.

It is only in the last decade that 3D sequences have become widely available. Our method was performed using a commercially available PACS and 3D reformation plug-in in one-Vendor (IMPAX EE R20 VXII SU4, Agfa HealthCare N.V.), and in our opinion appears to be applicable in any routine clinical PACS environment without dedicated postprocessing tools.

Although rater 1 missed 3 microcephaly and 3 macrocephaly patients, rater 2 falsely classified 6 and 3, respectively, and rater 3 rated 8 macrocephalies as normal HC, the accuracy was high. Thus, we would consider the MRI-based measurement reliable and fast (postprocessing time < 1min). A high interrater agreement also supports this conclusion [16]. More than the ICC, the TEM is a central parameter in anthropometric measurements, as the ICC does not account for bias in measurement [20]. Thus, results of the TEM show a more differentiated picture. While the TEMs of readers 1–2 and readers 1–3 were acceptable, the relative TEM of readers 2–3 only reached 1.6%. According to the current literature, trained readers should reach a relative TEM of 1.5% and below [18, 21]. Readers in this study were trained neuroradiologists; however, as this study assessed a new measurement for the assessment of the HC, we would still consider all TEMs of acceptable quality. Accordingly, it can be assumed that the MRI-based HC measurement yields approximately reproducible and reliable HC values as the clinical tape measurement [22]. With respect to the missed pathological HC, it should be mentioned that these were borderline percentiles.

Compared to previous studies, our study has the strength that we included a high number of pathological HC cases.

However, this study shows some limitations including the number of patients and the time period between clinical HC measurement and imaging. The retrospective design did not allow a recheck of the clinical measurements, and thus incorrect clinical measurements may occur in the data. The age range was limited to younger patients since HC measurement are not commonly acquired in adolescence. Additionally, due to the monocentric design an inherent risk of bias appears. The fact that the tape measurement HC values were revealed to the raters after the first nine measurements had no relevant effect on the results, since the MRI-based measurements were not allowed to be changed.

## Conclusion

This study implies that the MRI-based measurement of the head circumference provides a reliable approximation of the established clinically measured head circumference. The increasing availability of 3D datasets presumably allows the simple reproduction of clinical measurements of the outer circumference of the head. Thus, this method of HC measurement can be very useful in both clinical and research settings.

## Abbreviations

HC: Head circumference
ICC: Intraclass correlation coefficient
ROI: Region of interest
TEM: Technical errors of measurements
